# Maternal exposure to dim light at night induces behavioral alterations in the adolescent and adult offspring Wistar rat

**DOI:** 10.3389/fphys.2024.1520160

**Published:** 2025-01-07

**Authors:** Shellye González-González, Mariana Gutiérrez-Pérez, Mara A. Guzmán-Ruiz, Estefania Espitia-Bautista, Rosa María Pavón, Karla P. Estrada-Rodríguez, Alejandro Díaz-Infante R., Cecilia G. Guadarrama Gándara, Carolina Escobar, Natalí N. Guerrero-Vargas

**Affiliations:** 1 Departamento de Anatomía, Facultad de Medicina, Universidad Nacional Autónoma de México, Ciudad de México, Mexico; 2 Department of Psychology, University of Fribourg, Fribourg, Switzerland; 3 Departamento de Fisiología, Facultad de Medicina, Universidad Nacional Autónoma de México, Ciudad de México, Mexico; 4 Instituto Nacional de Psiquiatría, Ramón de la Fuente Muñiz, Ciudad de México, Mexico; 5 Facultad de Arquitectura, Universidad Nacional Autónoma de México, Ciudad de México, Mexico

**Keywords:** circadian disruption, dim light at night, offspring, social play behavior, nucleus accumbens, microglia, depressive and anxiety-like behaviors

## Abstract

**Introduction:**

Access to electric light has exposed living organisms to varying intensities of light throughout the 24 h day. Dim light at night (DLAN) is an inappropriate signal for the biological clock, which is responsible for the circadian organization of physiology. During the gestational period, physiological adaptations occur to ensure a successful pregnancy and optimal fetal development. Environmental maternal conditions, such as disruptions of maternal circadian rhythms, could negatively affect offspring health. We have previously demonstrated that exposure of female Wistar rats to DLAN results in circadian, metabolic, and behavioral alterations. A relevant behavior during adolescence is social play, primarily regulated by the nucleus accumbens (NAc) which is crucial for the proper performance of important behaviors in adulthood. Throughout development, microglia are responsible for the remodeling of diverse brain regions via synaptic pruning. During adolescence, this process occurs within the NAc, where immune-mediated remodeling directly impacts social play behavior.

**Methods:**

This study investigated the effects of maternal exposure to DLAN or a light-dark cycle (LD) before (5 weeks) and during the gestational period (21–23 days) on the metabolism and behavior of offspring in adolescence and adulthood. Body mass was measured every 5 days from postnatal day 1 (PN1) to PN25 and every 10 days from PN40 to PN90; food consumption was monitored weekly from PN40 to PN90. Social play behavior was evaluated at PN40. The quantification and morphology of microglia in the NAc were measured on PN30. An open field test was conducted at PN60, and anhedonia test was assessed at PN90.

**Results and discussion:**

Male and female offspring from mothers exposed to DLAN showed increased body mass gain at PN25. DLAN male offspring had lower food consumption, while DLAN females exhibited increased food consumption. In social play behavior, no differences were found between DLAN and LD female offspring. In contrast, DLAN male offspring exhibited a significant decrease in social play behavior compared to LD animals, which was associated with higher numbers of microglia in the NAc that had more ramified morphology. Importantly, at PN90, DLAN offspring presented increased anxiety-like behaviors. These results demonstrate that DLAN exposure induces intergenerational behavioral alterations that persist until adulthood.

## 1 Introduction

The circadian system regulates the temporality of physiological and behavioral functions enabling organisms to anticipate and adapt to the changing conditions of the environment (Pilorz et al., 2018). The alternation of the light-dark cycle is the main time cue that synchronizes the mammalian circadian system to the external 24 h day (Vetter, 2020). It is estimated that more than 80% of the population worldwide is exposed to nocturnal light (Falchi et al., 2016), which is considered by the World Health Organization as a social determinant of health, that is, a “non-medical factor that influences health outcomes” (WHO, 2022). This abnormal exposure to light during the night affects the organization of circadian rhythms and increase the risk of developing metabolic and emotional disorders like anxiety and depression (Navara and Nelson, 2007).

In our everyday environments, we are exposed to various types of artificial light at night each produced by different sources and varying in intensity. Street lamps, light bulbs, illuminated billboards, and windows of commercial buildings emit light that can enter through household windows (Chepesiuk, 2009), becoming a constant source of dim light at night (DLAN). Despite its low intensity (5–30 lux) (Walbeek et al., 2021), exposure to DLAN is associated with sleep disturbances in humans (Cho et al., 2016) and with disruption of the daily sleep-wake cycle (Stenvers et al., 2016), metabolic alterations (Borniger et al., 2014; Fonken et al., 2013), spatial memory impairments (Liu et al., 2022), and depressive and anxious-like behaviors in rodents (Cissé et al., 2016; Fonken and Nelson, 2013). We have previously demonstrated that exposing female Wistar rats to DLAN (5–7 lx) for 5 weeks induce the loss of activity and body temperature rhythms, metabolic alterations, low melatonin levels and irregular estrous cycles (Gutiérrez-Pérez et al., 2023), suggesting that DLAN exposure could have an impact on the reproductive and gestational capacity of female rats.

During the gestational period, physiological, endocrine, and immunological adaptations occur to ensure a successful pregnancy and optimal fetal development (Chang and Streitman, 2012). Environmental conditions experienced by the mother during gestation significantly impact the developing fetus (Muglia et al., 2022) increasing its vulnerability to chronic diseases later in life (Barker, 2007). This concept is referred to as the developmental origins of health and disease (DOHaD) (Gluckman et al., 2010). Maternal circadian disruption caused by exposure to light at night or changes in the photoperiod, that occur during pregnancy, negatively affects offspring health (Mendez et al., 2016) through mechanisms that have not been determined. However, recent evidence support the role of epigenetic modifications, oxidative stress, melatonin and corticosterone programming (Hsu and Tain, 2020). In primates, constant bright light conditions during the gestational period impair the body temperature rhythms of the offspring (Serón-Ferré et al., 2013). In rodent models, circadian disruption during pregnancy induces a great variety of physiological and behavioral alterations such as: increased body mass, increased adiposity, enhanced response to intraperitoneal glucose (Halabi et al., 2021), cardiac hypertropia (Galdames et al., 2014), disrupted daily plasma melatonin (Mendez et al., 2016) and metabolic rhythms (Dzirbíková et al., 2022), as well as the development of anxiety-like behaviors (Borniger et al., 2014).

The development of behavioral disorders in the offspring can lead to depression and anxiety during adulthood (Borniger et al., 2014) which have been associated with alterations in synaptic plasticity processes that take place during critical developmental periods (Marsden, 2013).

In rats, an important behavior during adolescence is social play behavior, which is characterized by a series of standardized motor patterns resembling functional behaviors observed in later stages of development, such as sexual behavior, predation, and aggressive peer interactions (Pellis and Pellis, 2017; Panksepp, 1981; Thor and Holloway, 1984). Social interactions during play behavior are critical for social and cognitive development in adulthood (Pellis et al., 2022). In males, but not in females, it has been identified that the dopaminergic pathway in the nucleus accumbens (NAc) that plays an important role in the expression of play behavior is refined through synaptic pruning, mediated by microglial phagocytosis of dopamine 1 receptor (D1r) mainly at PN30. Preventing microglial phagocytosis alters the expression of social play behavior suggesting that the remodeling exerted by microglia during adolescence is crucial for the proper development of social play behavior (Kopec et al., 2018).

This study aims to contribute to the DOHaD framework by investigating the intergenerational effects of maternal circadian disruption, due to DLAN exposure on metabolism and behavior of the offspring during adolescence and adulthood. In addition, the expression of the microglial marker IBA-1 was evaluated in the NAc of the adolescent male offspring. Finally, we also analyzed the long-term behavioral effects of maternal DLAN exposure by evaluating anxiety and depressive-like behavior in the adult offspring.

## 2 Materials and methods

### 2.1 Animal and general housing conditions

Adult female Wistar rats (n = 35), weighing 200–220 g (7–8 weeks old) and male Wistar rats (n = 10; 300–350 g, 10–12 weeks old), were obtained from the *Unidad Académica Bioterio* of the Facultad de Medicina, Universidad Nacional Autónoma de México (UNAM). Animals were individually accommodated in transparent acrylic cages measuring 40 cm × 50 cm × 20 cm, positioned atop tilt sensors. The cages were placed within soundproof, well-ventilated activity monitoring system, that can house up to 8 cages each with controlled ambient temperature (22°C ± 1°C), consistent airflow, and controlled illumination conditions of 12:12 h light-dark cycle (LD; 12 h, 200–250 lx: 12 h, 0 lx) where lights on were set at 7:30 h, defined as Zeitgeber time 0 (ZT0), while lights off were set as 19:30 h, defined as Zeitgeber time 12 (ZT12). All rats were left unmanipulated for 7 days for habituation. Nutritional needs were met through *ad libitum* access to Rodent laboratory chow (Purina 5001, Purina, Minnetonka, MN, United States) and fresh water. All rats remained inside the monitoring system for the entire protocol and only illumination patterns were modified as will be described in the next section.

Experiments were approved by the committee for research and ethical evaluation at the Facultad de Medicina, UNAM (FM/DI/070/2023). The use of animals was approved by the “*Comité Interno para el Cuidado y Uso de los Animales de Laboratorio*” from the Facultad de Medicina, UNAM (023-CIC-2023) and in strict accordance with the Mexican norms for animal handling Norma Oficial Mexicana NOM-062-ZOO-1999, which conforms to international guidelines for animal handling. All efforts were made to minimize the number of animals and their suffering.

### 2.2 Experimental design

During the baseline (BL) rats were housed in individual cages in the activity monitoring system and left for 7 days undisturbed in LD conditions. Male rats were only used for reproductive purposes and hence were maintained in LD conditions. Female rats were randomly assigned to one of two conditions: 1. light-dark cycle mothers (LDm, n = 10; 12 h, 200–250 lx, 3730K:12 h, 0 lx) or 2. dim-light exposure at night mothers (DLANm, n = 10 12 h, 200–250 lx, 3730K lx:12 h 5–7 lx,1638K). Light was supplied using SHOME-120 LED RGB + W 10W light bulbs (Steren, China). Illumination and color temperature were measured within each habitat cage using a Sekonic C-800 spectrometer (Sekonic, Tokio, Japan) (Supplementary Figure S1). Female rats remained in the previously assigned condition for 5 weeks. At the beginning of week 6, rats from each group were paired with one male rat at a ratio of 2:1 for mating purposes for 1 week. The lighting condition for each group was maintained during this mating week, pregnancy confirmation and throughout the gestational period (21–23 days). Pregnancy was confirmed by the presence of sperms visualized in the vaginal smears performed daily during the mating week. The day in which sperms were identified was defined as gestational day 0. Females without sperms in the vaginal smears (LDm n = 5; DLANm n = 10) were excluded from the study.

Pairing resulted in two groups:

1. LD offspring: male and female pups from mothers maintained in LD conditions throughout the entire protocol.
2. DLAN offspring: male and female pups from mothers exposed to dim light at night before (5 weeks), and during mating (1 week) and during pregnancy period (21–23 days).

Importantly, in postnatal day 0 (PN0), the mothers and their pups independently of their previous condition were placed under LD conditions. On PN1, the offspring were sexed, and litters were adjusted to contain 10 pups per litter (five males and five females), each group of LD or DLAN pups contained animals from at least three different litters (Supplementary Table S1). The pups were weaned on PN21 and group-housed with same sex siblings. We developed two series of experiments, in each series five LDm and five DLANm were included.

The maternal behavior was evaluated in the mothers of both series on PN10. Weight gain (PN1-PN90), food ingestion (PN40-PN90), and social play behavior (PN40) were measured in female and male offspring of both series.

**FIGURE 1:**
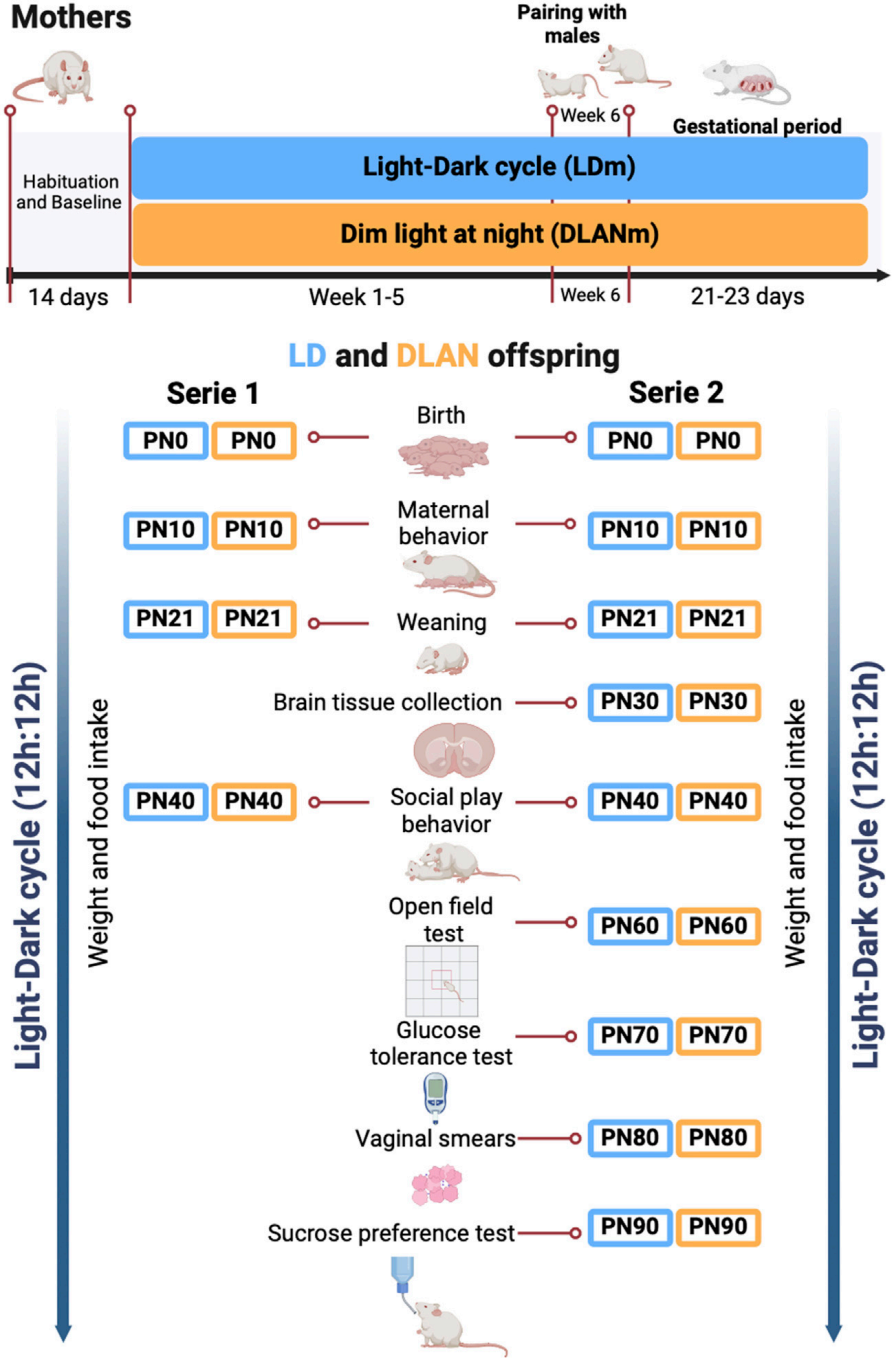
The experimental design. Female rats were randomly assigned to the experimental groups. Light–dark mothers (LDm) and dim light at night mothers (DLANm). These mothers gave birth to LD offspring (male and female pups from mothers maintained in LD conditions all the protocol) and DLAN offspring (male and female pups from mothers exposed to dim light at night before (5 weeks), during mating and pregnancy period. At postnatal day 0 (PN0), mothers and their pups independently of their previous condition were placed under LD conditions.

In the second series of male and female LD and DLAN offspring besides the already mentioned evaluations, LD and DLAN male (n = 4/group) offspring were euthanized at PN30 for brain tissue collection and subsequent immunofluorescence to evaluate the number of IBA-1 positive cells and microglial morphology in the NAc. The rest of the animals were evaluated as follows: on PN60 depressive and anxiety-like behaviors were assessed, on PN70 a glucose tolerance test was performed, from PN80-PN88 the estrous cycle of the female offspring was determined, and finally, a sucrose preference test was performed on PN90 in both males and females (Figure 1).

### 2.3 Maternal behavior

Maternal behavior was evaluated on PN10 at ZT6 due to the reported higher frequency of this behavior at this time of the day (Capone et al., 2005). The test was carried out in a noise-free room. The mothers (n = 10/group) were initially separated (individually housed in a separate acrylic cage) from their offspring for 30 min (Capone et al., 2005). Subsequently, the offspring were individually placed and spaced apart inside the cage. Immediately after, the mother was introduced into the cage for 5 min. During this time, interactions were video recorded with a smartphone camera. The videos were reviewed by two blind observers who documented the continuously occurrence of the following behaviors:

1. Carrying: When the mother held the offspring in her mouth to move them from one space within the cage to another.
2. Licking: When the mother licked her offspring by passing her tongue over them.
3. Latency of maternal behavior: the time it takes for the first maternal behavior to occur.

### 2.4 Metabolic measurements

#### 2.4.1 Body mass gain and food intake

Body mass was monitored every 5 days from PN1 to PN25 in males (n = 18–24/group) and females (n = 20–21/group), and then every 10 days from PN40 to PN90 in males (n = 13–14/group) and females (n = 9–13/group) (Supplementary Figure S2). Rats were not disrupted for body mass measuring during the social play behavior evaluations from PN30-PN40. Body mass gain was determined by subtracting the initial body mass from the final body mass.

From PN1 to PN25, each rat was individually marked to facilitate identification and enable the monitoring of weight fluctuations while they were allocated with their litters. Measurements were performed at the same time of the day (ZT6) on Tuesdays. 24 h food ingestion was monitored every 10 days from PN40 to PN90 in males (n = 13/group) and females (n = 9–12/group). For this, 50 g of chow 5001 food was measured and placed in each animal’s cage at ZT0, 12 h later (ZT12) the remaining food was measured and replaced with another 50 g and repeated this procedure 12 h later. These measurements were performed in all the pups.

#### 2.4.2 Glucose tolerance test (GTT)

At PN70, a GTT was performed as described in (Gutiérrez-Pérez et al., 2023). Briefly, after 16 h of overnight fasting a basal blood sample was obtained from the tail vein at ZT0 in males (n = 7/group) and females (n = 7/group), and an intraperitoneal injection of 1 g of glucose/kg in 0.9% saline solution was immediately given. After glucose administration, subsequent blood samples were collected from tail puncture (0, 15, 30, 60 and 120 min respectively). Glucose levels were determined with a blood glucose monitor (Elite, Bayer, Elkhart, IN, United States) and glucose strips Ascencia Elite (Bayer, Basel, Switzerland).

### 2.5 Estrous cycle determination

To determine the stage of the estrous cycle, vaginal cytology was conducted as described in (Gutiérrez-Pérez et al., 2023). Briefly, a vaginal wash was performed daily at ZT3 over a period of eight days from PN80 to PN88 (n = 10/group). 60 μL of sterile saline solution were flushed into the vaginal cavity. Collected vaginal smear was stained with H&E and observed at the microscope. The estrous cycle stage was determined by identifying the predominant cell type in each sample as previously reported (Cora et al., 2015; Marcondes et al., 2002). Regular cycles last from 4 to 5 days (1 proestrus day, 1 estrous day and 1 diestrus I day and 1-2 diestrus II days) and they must have the sequence mentioned before.

### 2.6 Behavioral measurements

#### 2.6.1 Social play behavior

This test was carried out at PN40 in males (n = 14–15/group) and females (n = 13–14/group), a developmental stage corresponding to mid-adolescence (Kopec et al., 2018), which is marked by a notable presence of playful behavior (Panksepp, 1981). The test was conducted between ZT12 and ZT13, a period of increased play behavior activity (Panksepp, 1981). The room was illuminated with a 5W-127V red light (<5 lx) in a noise-free environment. Each tested rat was isolated for 24 h prior to the test to motivate play behavior. After isolation, 5 rats of the same sex and different litters (at least from 3 different litters) were placed in a new cage, and a 5 min video was recorded with a smartphone camera to subsequently assess the following social play behaviors:

1. Pinning: When one rat partially or fully positions itself over another rat in a supine posture.
2. Pouncing: When one rat partially or completely positions itself on the back of another rat.
3. Boxing: When two rats face each other and initiate pushing motions with their forepaws.
4. Chasing: When one rat pursues another rat.

The continuous occurrence of these behaviors was counted during the 5 min recording. Total play behavior was calculated as the sum of all previously mentioned behaviors per animal. Behavioral assessments were done double blinded by two independent observers.

#### 2.6.2 Open field test (OFT)

The OFT was performed in male (n = 8/group) and female (n = 8–9/group) offspring at PN70 between ZT12 and ZT13 using an acrylic square arena (59.3 cm wide × 59.5 cm log) divided into 25 squares. The room was illuminated with a 5W-127V red light (<5 lux). To start the test, each rat was placed in the right corner of the open field and the spontaneous activity was recorded with a smartphone camera for 5 min. After testing each rat, the open field was cleaned with a 70% alcohol solution. From the video recordings behaviors used as indicators of anxiety-like behavior were counted: rearing events, grooming events and number of crosses to the center. Behavioral assessments were done double blinded by two independent observers.

#### 2.6.3 Sucrose preference test

To determine the presence of depressive-like behaviors in the adulthood (PN90) LD and DLAN male offspring (n = 10/group) and female offspring (n = 9/group) were exposed for one night before the test, to a 5% sucrose solution instead of tap water for 24 h to avoid neophobia to the sucrose solution during the test. The night before the test rats were deprived of tap water for 12 h, the following day, at ZT12, two bottles were placed in their home cages for 30 min, one with 50 mL of 5% sugar water the other with 50 mL of tap water. We measured the amount of sucrose solution and tap water consumed in mL. The preference for sugar water was determined as an index of discrimination (DI) (%): mL of sucrose solution × 100/ (mL water + mL sucrose solution). Rats will normally prefer to drink sweet water as it is rewarding, and an anhedonic subject does not present this preference (Tchekalarova et al., 2018). At the end of the test, rats were allowed free access to tap water.

### 2.7 Immunofluorescence

On PN30, the male offspring of the second series of mothers (n = 4/group) were euthanized by injecting an overdose of sodium pentobarbital i.p. (PiSA, 65 mg/kg). When the animals were deeply anesthetized and shortly before cardiac arrest, we initiated the intracardial perfusion of 9% saline solution for 5 min (perfusion rate 15 mL/min), followed by 5 min of 4% paraformaldehyde (PFA) in phosphate-buffered saline (PBS). The brains were collected, and post-fixed in 4% PFA for 48 h and cryoprotected in 30% sucrose, 0.05% sodium azide in PBS and stored at 4°C until use. Serial 40 μm thick coronal sections were obtained by using a Leica CM1850 UV cryostat. The Shell and Core areas of the Nucleus Accumbens (NAc; 1.60 mm and 9.20 mm posterior to bregma) were identified according to the Atlas of the rat brain in stereotaxic coordinates for the postnatal period (Khazipov et al., 2015). Sections were stored at 4°C until immunofluorescence (IF) processing. Three slices containing the anterior, medial, and posterior NAc per animal were pre-incubated with 1% H_2_O_2_ solution for 10 min to block the endogenous peroxidase activity. After a brief wash with 1% PBS, the sections were co-incubated overnight at room temperature with constant stirring with the primary antibody to IBA-1 (Abcam, ab5076, 1:1000). The next day, the sections were washed with PBS and co-incubated for 2 h with a fluorescent secondary antibody Alexa Fluor 647 anti-goat (Jackson 128-607-232, 1:500), after this incubation sections were washed with PBS and placed on gelatin coated slides.

After drying, the slides were incubated for 5 min with DAPI solution (Invitrogen, 4′,6-diamidino-2-phenylindole, dihydrochloride, D1306) and covered with 70 μL of anti-fade fluorescence mounting medium (Abcam, ab104135). Two photomicrographs (one of the right side and one of the left side) from 3 slices of each animal (4/group) were obtained with a ZEISS LSM 880 confocal microscope at 40×. Subsequently, integrated density, cell count and sholl analysis were performed for IBA-1 using the Fiji free software (Schindelin et al., 2012).

### 2.8 Cell count analysis of IBA-1 positive cells

To measure the number of IBA-1 positive cells (macrophage marker), confocal images were split into their channel components using Fiji software (Schindelin et al., 2012). Subsequently, the DAPI channel was duplicated to serve as a reference, and then a merge was generated between the IBA-1 channel and original DAPI channel. Then by using the *Multi-point* function we marked only the IBA-1 + DAPI positive cells present in the NAc Core and Shell regions.

### 2.9 Sholl analysis

Microglia process arborization in the NAc were quantified by using the Fiji Sholl analysis to determine number of intersections between microglial branches and each Sholl ring. We evaluated three NAc slices of four animals from each experimental group, where three random microglial cells were processed from the left and right NAc Core and three from the NAC Shell. The individual cells were transformed into binary images of the IBA-1 corresponding channel, through the *Threshold* command a threshold was set until the branches were clearly visible. Subsequently, any noise was manually removed, and the Sholl Analysis plugin from Neuroanatomy (Ferreira et al., 2014) was used to measure the number of microglial branches intersecting each circle (2 µm apart around the center of the cell body) to create a Sholl curve for each cell, these curves were averaged to build the curve for each animal as previously reported by (Liu et al., 2022).

### 2.10 Statistical analysis

Data were presented as mean ± the standard error of the mean (SEM). Values were tested for normality using the Kolmogorov-Smirnov Z test. Maternal behaviors carrying and licking, social play behaviors and data from the OFT were evaluated using a two side, non-parametric Mann-Whitney Test, assuming equal variables. The latency to present maternal behaviors and the number of cells positive to IBA-1 were evaluated with a Student’s *t*-test. Fisher exact test was used for comparison of percentage of females with regular or irregular estrous cycles. Weight gain and food intake were analyzed with a two-way RM ANOVA for two factors: postnatal days x experimental condition. The GTT was analyzed with a two-way RM ANOVA for two factors: time after glucose x experimental condition (LD or DLAN). Microglial intersections were analyzed with a two-way RM ANOVA for two factors: experimental condition (LD or DLAN) x distance from cell body. All the two-way ANOVA analysis were followed by a Sidak’s multiple comparison *post hoc* test. Significance was defined as *p* < 0.005. All data were analyzed and visualized using GraphPad Prism (version 10; Graph Pad Software, Inc.).

## 3 Results

### 3.1 Maternal exposure to DLAN leads to metabolic disruptions in body mass gain and food consumption in the offspring

**FIGURE 2:**
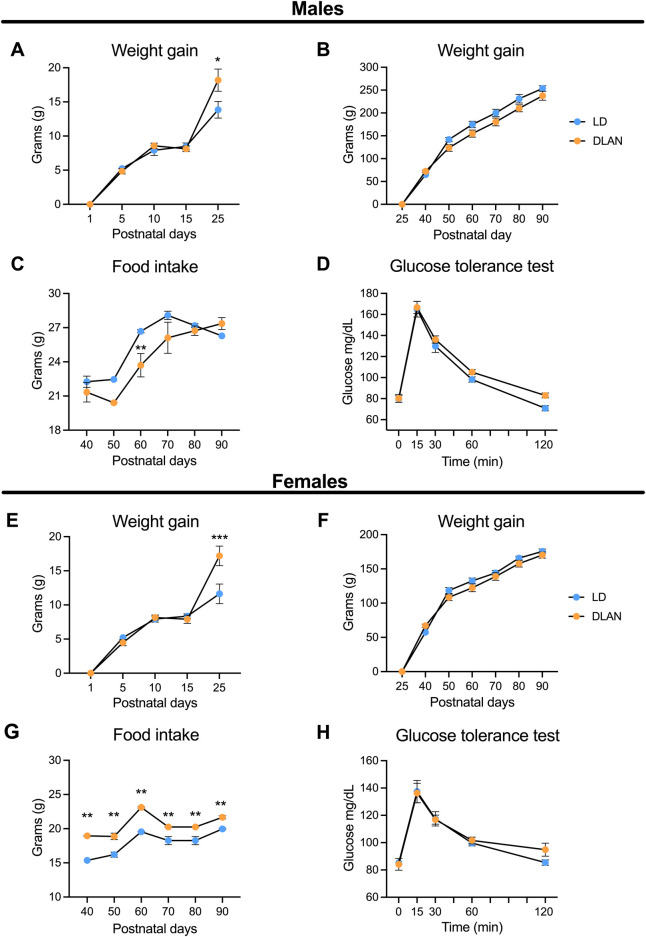
Maternal DLAN exposure increases weight and food intake in both male and female offspring. **(A)** Weight gain of male offspring from mothers maintained in a regular light-dark cycle (LD, blue circles; n = 18) and male offspring of mothers exposed to dim light at night (DLAN, orange circles; n = 24) from postnatal day 1 (PN1) to PN25. **(B)** Weight gain of male LD and DLAN offspring from PN40 until PN90 (n = 13 per group). **(C)** Food intake of male LD and DLAN offspring from PN40 to PN90 (n = 13/group). **(D)** Glucose tolerance test (GTT) of male LD and DLAN offspring (n = 6–7/group). **(E)** Weight gain of female LD (blue circles; n = 20) and DLAN offspring (orange circles; n = 21) from PN1 to PN 25. **(F)** Weight gain of female LD and DLAN offspring from PN40 to PN90 (n = 9–12/group). **(G)** Food intake of female LD and DLAN offspring from PN40 to PN90 (n = 9–12/group). **(H)** GTT between female LD and DLAN offspring in PN70 (n = 7/group). Data is shown as the mean ± SEM. The Sidak’s *post hoc* test indicated significant differences between groups *p < 0.05, **p < 0.01, ***p < 0.001.

To determine if maternal DLAN affects metabolic parameters in the offspring we evaluated body mass gain and food consumption at different moments before and after weaning. DLAN male and female offspring exhibited high birth body mass as compared to LD offspring (Supplementary Table S1; males: t = 4.27, df = 40, *p* = 0.0001, females: t = 3.77, df = 41, *p* = 0.0005). Increased body mass gain was observed in both DLAN male and female offspring only at PN25, compared to LD offspring (Figures 2A, E). The two-way RM ANOVA indicated a significant interaction between postnatal days and experimental condition (males: *F*_(4, 160)_ = 3.35, *p =* 0.01; females: *F*_(4, 164)_ = 6.97, *p* < 0.0001). From PN40 to PN90 males and females from both groups maintained similar body mass gain (Figure 2B, *p* = 0.09; Figure 2F, *p* = 0.40).

In addition, we observed that male DLAN offspring decreased their food consumption at PN60 as compared to LD rats (Figure 2C). The two-way RM ANOVA indicated a significant interaction between postnatal days and experimental condition (*F*_(5, 120)_ = 3.54, *p* = 0.005). Interestingly, female DLAN offspring exhibited increased food consumption as compared to LD females, and this difference was observed from PN40 until PN90 (Figure 2G). The two-way RM ANOVA indicated a significant interaction between postnatal days and experimental condition (*F*_(5, 95)_ = 3.08, *p* = 0.01).

We also performed a glucose tolerance test (GTT) for both LD and DLAN male and female offspring. There were no significant differences in the plasmatic glucose levels between male (Figure 2D, *p* = 0.55) and female groups (Figure 2H, *p* = 0.67).

### 3.2 The estrous cycle of the female DLAN offspring is not disrupted

**FIGURE 3:**
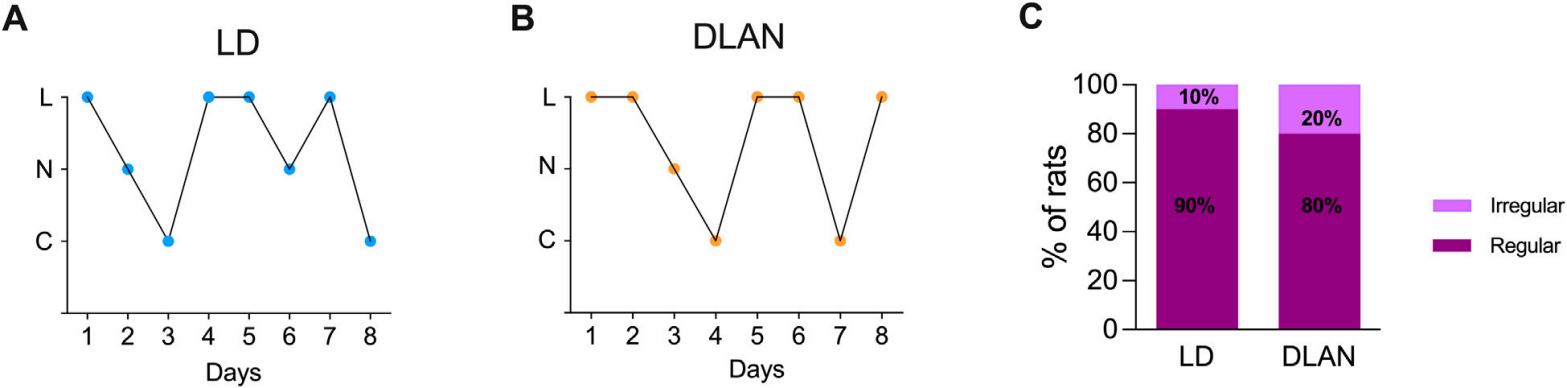
The estrous cycle of the female offspring from mothers exposed to DLAN was not affected. **(A)** Representative graph of the estrous cycle of a female rat from a mother maintained in a regular light-dark cycle (LD, blue circles; n = 8). **(B)** Representative pattern of a female rat from a mother exposed to dim light at night (DLAN, orange circles; n = 10). “L” refers to leukocytes, “N” to nucleated cells, and “C” to cornified cells. **(C)** Percentage of rats with regular and irregular estrous cycles in the LD and DLAN groups.

To determine whether maternal DLAN exposure alters female offspring estrous cycle, we evaluated vaginal smears from PN80-PN88. We observed that both DLAN and LD offspring exhibited normal estrous cycles (Figures 3A, B) with no significant differences in the percentage of regular versus irregular rats between groups (Figure 3C; Fisher’s analysis, p = 0.07).

### 3.3 Maternal exposure to DLAN diminish social play behavior in male offspring but not in female offspring

**FIGURE 4:**
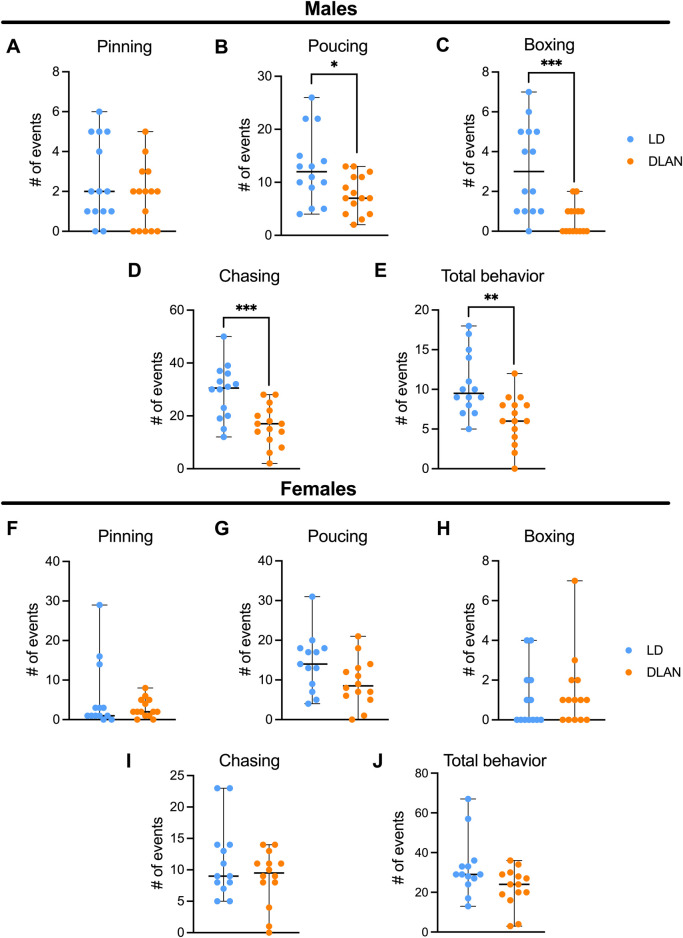
Maternal DLAN exposure decrease social play behavior in male offspring, but not in females. Number of events of **(A)** pinning, **(B)** pouncing, **(C)** boxing, **(D)** chasing events and **(E)** total play behaviors of male offspring from mothers exposed to a light-dark cycle (LD, blue circles; n = 14) and male offspring from mothers exposed to dim light at night (DLAN, orange circles; n = 15). Number of events of **(F)** pinning, **(G)** pouncing, **(H)** boxing, **(I)** chasing and **(J)** total play behaviors of female offspring from mothers exposed to a light-dark cycle (LD, blue circles; n = 13) and female offspring of mothers exposed to dim light at night (DLAN, orange circles; n = 14). Data are expressed as the median ± range. The Mann-Whitney test indicated significant difference among groups *p = 0.02, **p = 0.0020, ***p = 0.0005, ***p = 0.0009.

To determine if maternal DLAN exposure alters offspring behavior during adolescence, we evaluated the social play behavior in the LD and DLAN male and female offspring at PN40. Male DLAN offspring did not exhibit differences in pinning behavior as compared to male LD offspring (Figure 4A, *p* = 0.39). In contrast, a significant decrease in pouncing (Figure 4B, *p* = 0.02), boxing (Figure 4C, *p* < 0.01) and chasing behaviors (Figure 4D, *p* < 0.01), as well as in the total play behaviors (Figure 4E, *p* < 0.01) were observed in male DLAN offspring as compared to male LD offspring.

For the female offspring there were no differences between LD and DLAN animals in pinning (Figure 4F, *p* = 0.62), pouncing (Figure 4G, *p* = 0.18), chasing (Figure 4H, *p* = 0.77), boxing (Figure 4I, *p* = 0.46) and total behaviors (Figure 4J, *p* = 0.1885).

### 3.4 The NAc of male offspring of mothers exposed to DLAN presents more IBA-1 positive cells

**FIGURE 5:**
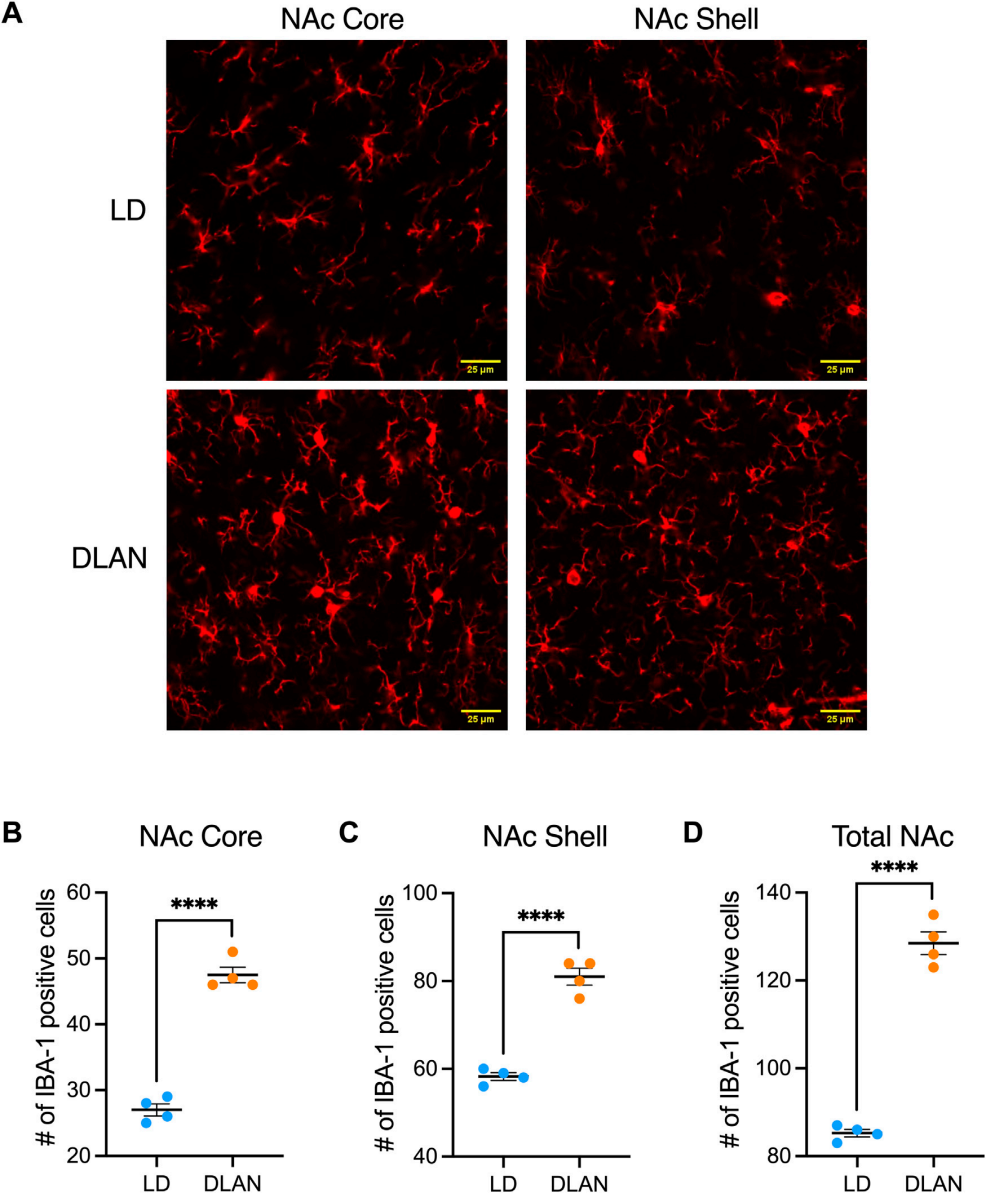
Number of positive cells for the microglial marker IBA-1 in the Core and Shell regions of the Nucleus Accumbens (NAc). **(A)** Representative confocal microscope images (40X) for the IBA-1 marker in the Core and Shell regions of the NAc of male offspring from mothers exposed to a light-dark cycle (LD, blue circles) and male offspring from mothers exposed to dim light at night (DLAN, orange circles). Number of IBA1-positive cells in the **(B)** Core, **(C)** Shell and **(D)** entire NAc (Core + Shell) of the LD and DLAN groups. Data are presented as the mean ± SEM; n = 4. Student’s t-test indicated significant differences among groups ****p < 0.0001.

In male rats, during adolescence (PN20-PN40), microglia are responsible for the pruning of the dopamine D1 receptors (D1r) in the NAc, which is an important mechanism for the development of social play behavior that is not observed in female rats (Kopec et al., 2018). For this reason, and because we did not observe significant differences in social play behaviors presented by LD vs. DLAN female rats, we only analyzed the brains of the male offspring. We observed a significant increase in the number of IBA-1 positive cells in the NAc Core and NAc Shell (Figure 5A), of the DLAN offspring as compared to the LD group (Figure 5B; NAc Core: t = 13.67, df = 6, *p* < 0001; Figure 5C; NAc Shell: t = 10.85, df = 6, *p* < 0.0001). Moreover, the IBA-1 positive cell count analysis of the entire NAc (Core + Shell) revealed an increased number of IBA1-positive cells in the brains of DLAN offspring as compared to the LD group (Figure 5D; t = 15.81, df = 6, *p* < 0.000).

### 3.5 The IBA-1 positive cells of the DLAN offspring presented a branched morphology

**FIGURE 6:**
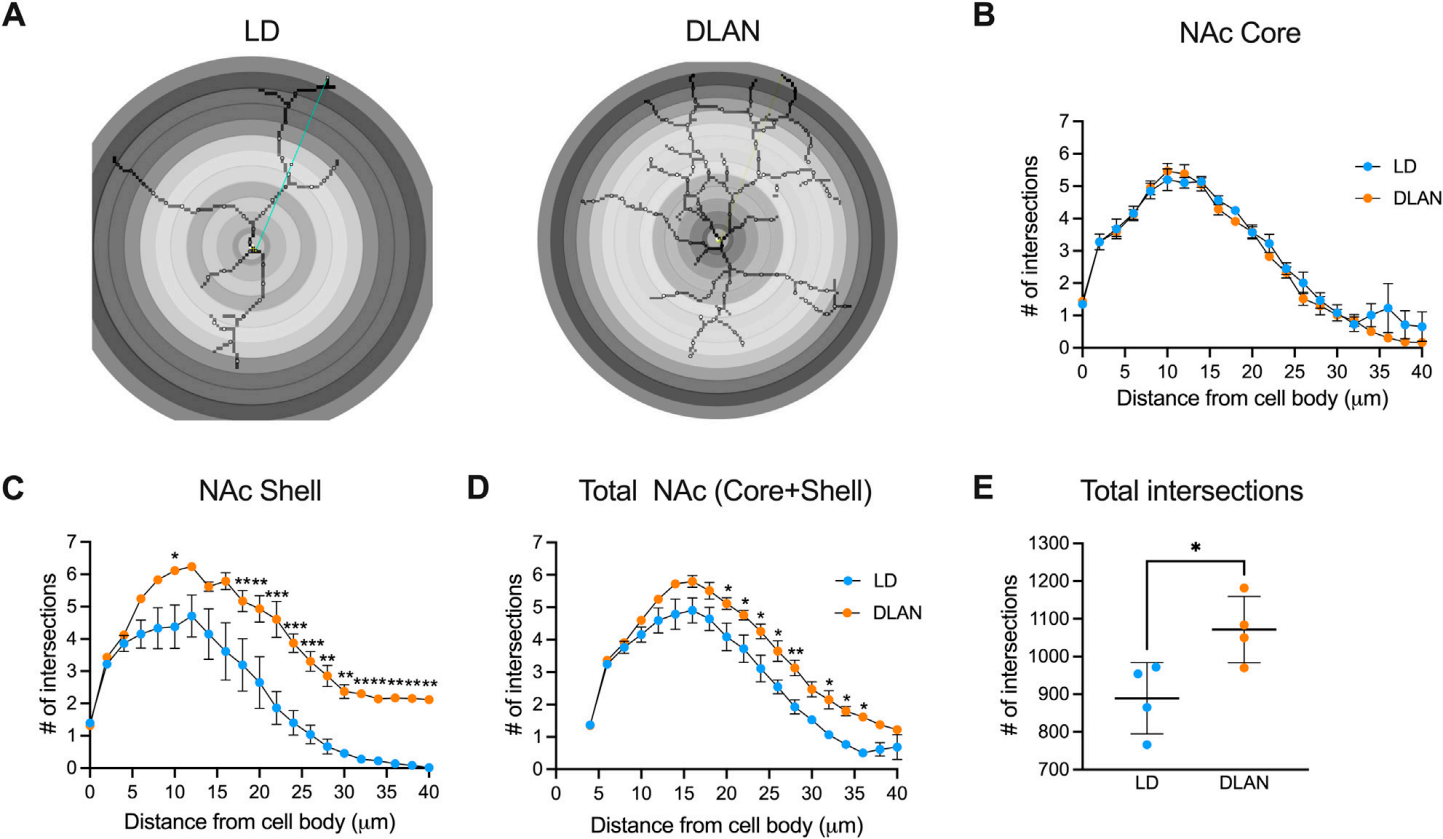
IBA-1 positive cells of male offspring from mothers exposed to DLAN exhibit a higher number of branch intersections in the Sholl analysis. **(A)** Schematic diagram of Sholl analysis for microglia in the NAc of male offspring from mothers exposed to a light-dark cycle (LD) and male offspring from mothers exposed to dim light at night (DLAN). **(B)** Number of intersections for LD (blue circles) and DLAN (orange circles) animals in the NAc Core **(C)**. Number of intersections in the NAc Shell of LD and DLAN offspring. **(D)** Number of intersections in the entire NAc (Core + Shell) for LD and DLAN offspring. **(E)** Total numbers of microglia branch intersections in the LD and DLAN group. Data is shown as the mean ± SEM. For **(B–D)**, the Sidak’s *post hoc* test indicated significant differences between groups *p < 0.05, **p < 0.01, ***p < 0.001.

Microglia undergoes morphophysiological transitions that reflect changes in their function (Morrison and Filosa, 2013; Fernández-Arjona et al., 2017). To further assess this, we analyzed microglial branch intersections in the NAc of LD and DLAN animals (Figure 6A). The results from the Sholl analysis reveal similar number of microglial branch intersections between IBA-1 positive cells of the NAc Core of LD and DLAN groups (Figure 6B, *p* = 0.77). In contrast, in the NAc Shell, microglia of the DLAN offspring showed an increased number of intersections at 16–40 mm from the center of cell body as compared to the LD group (Figure 6C, *p* < 0.05). In the entire NAc (including Core + Shell) IBA-1 positive cells of the DLAN offspring also showed increased number of intersections at 20–36 mm from the center of cell body as compared to the LD group (Figure 6D, *p* = 0.01). The two-way RM ANOVA indicated a significant interaction between experimental condition (LD or DLAN) × distance from cell body for the NAc Shell (*F*_(20, 120)_ = 4.8, *p <* 0.0001) and total NAc (*F*_(20, 120)_ = 2.17, *p =* 0.005).

In the entire NAc (including Core + Shell) the total number of microglia branch intersections were significantly increased in the DLAN group as compared to the LD group (Figure 6E *, p* = 0.03).

### 3.6 Maternal exposure to DLAN induces anxious-like behaviors in the adult offspring

**FIGURE 7:**
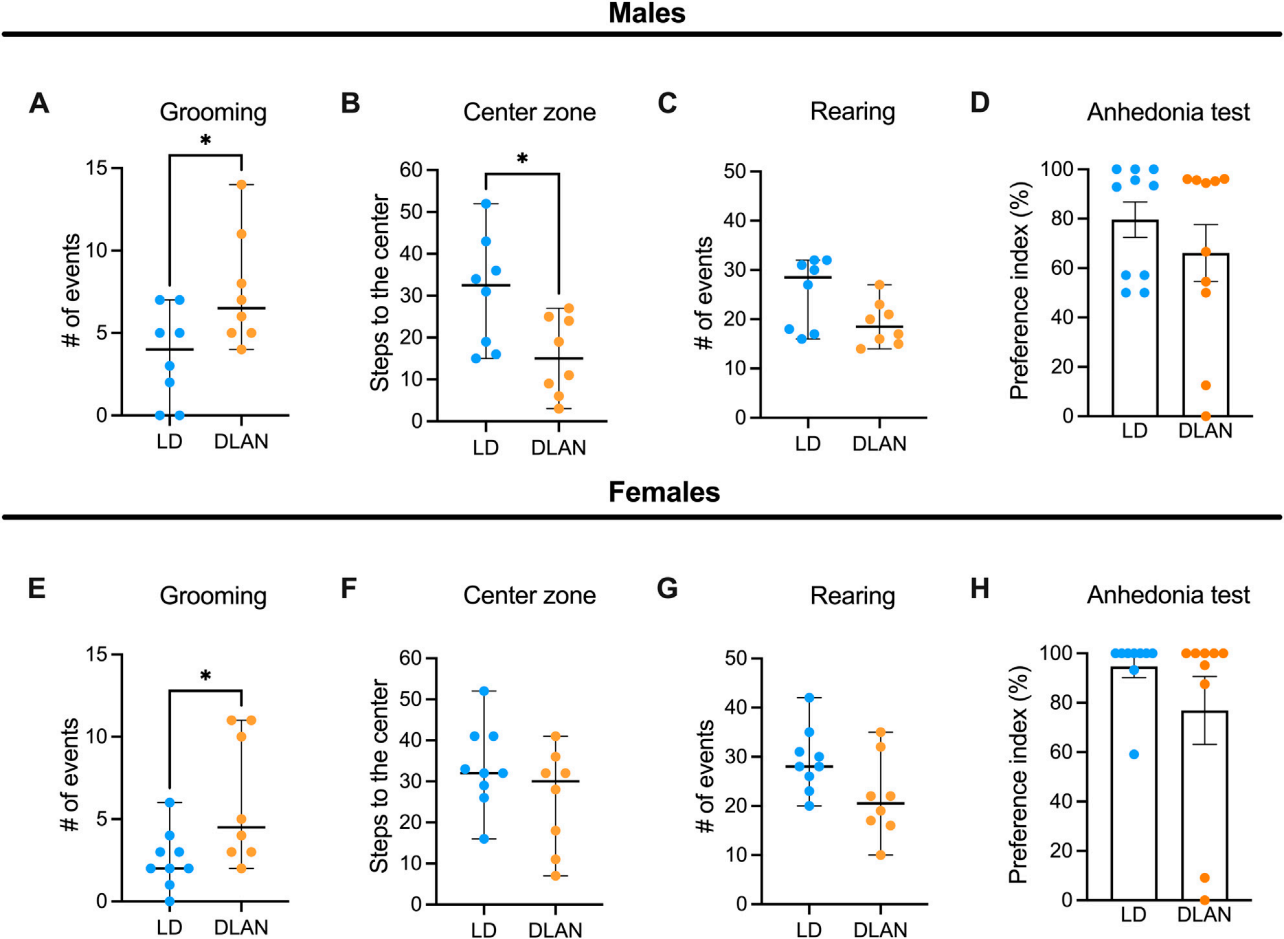
Maternal DLAN exposure increases anxiety-like behaviors in the adult offspring. **(A)** Number of grooming events, **(B)** steps to the center and **(C)** rearing events during the open field test (OFT) of male offspring of mothers exposed to a light-dark cycle (LD, blue circles; n = 8) and male offspring of mothers exposed to dim light at night (DLAN, orange circles; n = 8). **(D)** Preference index of sucrose ingestion in the anhedonia test (n = 10/group of male offspring). **(E)** Number of grooming events, **(F)** steps to the center and **(G)** rearing events during the OFT of female LD offspring (blue circles; n = 9) and female DLAN offspring (orange circles; n = 8). **(H)** Preference index of sucrose ingestion in the anhedonia test (n = 9/group). For **(A–C, E–G)** data are expressed as the median ± range. For **(D, H)** data are expressed as the mean ± SEM (n = 10–11/group). The Mann-Whitney test indicated significant difference between groups *p < 0.05.

Because we observed decreased social play behavior during adolescence which has been associated with long-term behavioral changes in adulthood (Walker et al., 2019), we evaluated depressive-like and anxiety-like behaviors in LD and DLAN offspring at PN90. In the open field test (OFT) male DLAN offspring exhibited increased number of grooming events (Figure 7A, *p* = 0.04) and decreased number of steps to the center zone of the open field (Figure 7B, *p* = 0.03) as compared to male LD male offspring. There were no differences in the number of rearing events between LD and DLAN male offspring (Figure 7C, *p* = 0.06). During the anhedonia test, we observed no significant differences in the sucrose preference between LD and DLAN animals (Figure 7D, *p* = 0.33).

Meanwhile, the female offspring of the DLAN mothers displayed a greater number of grooming events as compared to the LD offspring (Figure 7E, *p* = 0.03) but there were no significant differences in the number of crossings through the central squares (Figure 7F, *p* = 0.26) or in rearing behaviors between groups (Figure 7G, *p* = 0.06). Finally, no significant differences were detected in the sucrose preference index between LD and DLAN offspring (Figure 7H, *p* = 0.23).

Because maternal care can impact on behavioral development of the offspring (Curley and Champagne, 2016), we evaluated the number of carrying and licking events towards the offspring and the latency to exhibit maternal behavior in both LDm and DLANm mothers. We observed that DLAN exposure did not alter maternal behavior since DLANm and LDm presented the same amount of carrying and licking events. Furthermore, the latency to initiate the maternal behaviors was similar in the LDm and DLANm groups (Supplementary Table S2).

## 4 Discussion

The present study shows that maternal exposure to DLAN has intergenerational effects that vary in relationship to the offspring sex, with long-lasting behavioral consequences that can be detected until adulthood in both males and females.

Female offspring presented less intergenerational effects resulting from maternal DLAN exposure as we did not find any differences neither in the estrous cycle nor in social play behavior as compared to LD offspring. We have previously demonstrated that DLAN exposure arrests female rats in a persistent estrus state (Gutiérrez-Pérez et al., 2023). However, the intergenerational effects of DLAN on reproduction have only been described in flies, affecting mating and reproductive output (McLay et al., 2018). More studies are necessary to rule out the intergenerational effects of DLAN on reproduction. Maternal DLAN exposure did induce an increase in grooming behavior in the adult female offspring. Our findings are consistent with previous studies reporting that DLAN exposure during the gestational period leads to anxiety-like behaviors (Borniger et al., 2014).

Regarding the metabolic effects of maternal DLAN exposure, we observed that both offspring sexes exhibited high birth body mass and increased body mass gain at PN25. However, such differences were lost after weaning, suggesting a mother-dependent effect. It is known that the composition of maternal breast milk varies according to mothers health, diet, etc., reflecting the maternal physiological state (Marshall et al., 2022). Interestingly, while males showed reduced food intake, females exhibited increased food intake through development, suggesting sex-specific responses to maternal DLAN exposure. However, the absence of significant differences in the GTT and gain weight suggests that the intergenerational metabolic effects of DLAN might impact metabolism in a different manner, since recent studies indicate that gestational DLAN exposure disrupt metabolic rhythms of the offspring (Dzirbíková et al., 2022).

In the case of the male offspring, we observed that maternal exposure to DLAN induced a significant reduction in social play behavior. The expression of this behavior depends on a complex neuronal circuit known to process reward and motivation which includes structures such as the NAc (Lewis et al., 2021; Manduca et al., 2016). Studies have demonstrated that the remodeling of NAc occur during the rat adolescence where there is a burst in D1r expression in the same developmental stage in which social play behavior reaches its maximal peak of expression (PN30) (Kopec et al., 2018). The remodeling of this neuronal circuit is determined by microglial phagocytic activity, suggesting that these cells play a critical role in the establishment of the NAc neuronal network. We evaluated the NAc during adolescence, at PN30 and observed that this nucleus presented an increased amount of IBA-1 positive cells in the male offspring of DLAN mothers. Previous studies have demonstrated that DLAN increases microglial cells (IBA-1), pro-inflammatory cytokines, and induces oxidative stress in the hippocampus (Liu et al., 2022; Bedrosian et al., 2013). Increased microglial cells and morphological changes following DLAN exposure have been associated with synaptic loss, which, in turn, is linked to impaired spatial learning and memory in mice (Liu et al., 2022). In addition to its immune function, microglia play an important role in synaptic regulation (Tremblay et al., 2011) and their activation can interfere with synaptic plasticity processes specifically affecting social play behavior of male rats (Kopec et al., 2018).

Furthermore, the morphology of IBA-1 positive cells in the NAc of male DLAN offspring is more ramified. This morphology has been associated with the establishment of transient contacts with neurons and other glial cells (Nimmerjahn et al., 2005) and with indicators of phagocytosis of apoptotic cells and debris (Sierra et al., 2010; Kamei and Okabe, 2023). In line with our observations, the exposure of pregnant mice to Bisphenol A, induce a ramified microglial morphology with higher number of phagocytic cups (Rosin et al., 2022), suggesting that microglia might be affected by environmental factors to which the mothers are subjected during pregnancy with lasting effects into postnatal stages such as adolescence.

Changes in microglial morphology were particularly evident in the Shell region of the NAc. This region receives dopaminergic projections primarily from the VTA, a crucial pathway in the regulation of motivated and reinforcing behaviors (Salgado and Kaplitt, 2015) such as social play behavior. In contrast, the NAc Core region is innervated by projections from the substantia nigra, a region involved in motor control (Salgado and Kaplitt, 2015). This difference in the organization of projections suggests that the Shell and Core regions may differentially modulate reward behaviors. Our results suggest a possible relationship between the decrease in social play behavior and the increased amount of IBA-1 positive cells in the NAc. Future studies should assess if there is a particular vulnerability in the motivational dopaminergic circuits in response to maternal DLAN exposure. In addition, detailed measurements of microglial activity in the NAc and other brain areas involved in regulating social play behavior are needed.

Although no significant differences were observed in social play behavior between female DLAN and LD offspring, it is important to consider that social play behavior in females may differ in its manifestation compared to males (Kisko et al., 2021). Previous studies have suggested that females tend to display less aggressive and less intense play patterns compared to males (Thor and Holloway, 1984; Auger and Olesen, 2009). Therefore, this differential expression presents a methodological challenge, as the parameters used to assess social play behavior are primarily based on the standardized motor patterns observed in males, limiting the ability to observe gender variations and identify social play behavior patterns in females. In this sense, it is crucial to develop more specific approaches for evaluating motor social play behavior in females.

Moreover, we did not evaluate the NAc of the female offspring because previous studies have shown that synaptic pruning for social play behavior mediated by microglia during adolescence occur in males but not in females (Kopec et al., 2018). This suggests that other mechanisms, distinct from microglial-mediated synaptic pruning, may be involved in regulating synaptic development and social play behavior in females. Identifying these mechanisms is an important area for future research, as it could provide new insights into sex differences in neuronal and behavioral development in response to environmental factors such as DLAN exposure.

Dysfunctional social interactions during adolescence have been associated with long-term behavioral changes in adulthood (Vanderschuren et al., 2016). In line with this, we observed that maternal DLAN exposure induces increased anxiety-like behaviors in male offspring. Our results are in agreement with previous studies documenting the presence of anxiety-like behavior in the offspring of mothers exposed to DLAN during gestation (Borniger et al., 2014) and with depressive-like behaviors in the offspring of parents exposed to DLAN prior mating (Cissé et al., 2017). Interestingly, we also observed increased grooming behavior in the female offspring of DLAN exposed mothers. Overall, these results highlight the long-lasting effects on behavior of maternal DLAN exposure.

Maternal care influences offspring affective behaviors (Curley and Champagne, 2016; Kaffman and Meaney, 2007). In the present study we did not find differences in maternal care between DLANm and LDm mothers, suggesting that the observed effects in the offspring are not the result of alterations in maternal behavior during lactation. Instead, they may be related to physiological changes experienced by the mothers before or during pregnancy such as circadian disruption due to DLAN exposure (Gutiérrez-Pérez et al., 2023). Previous studies indicated that the deleterious effects of parental exposure to adverse environmental conditions can be transmitted to offspring (Breton et al., 2021; Hsu and Tain, 2020). Circadian disruption during gestation may influence offspring development through epigenetic modifications, such as DNA methylation, histone modifications, and non-coding RNA (Liu et al., 2024). In mice, circadian disruption induced by *jet lag* during pregnancy resulted in altered methylation of clock genes and hypermethylation of micro-RNA genes required for heart and bone development (Chaves et al., 2019).

Additionally, we previously demonstrated that DLAN exposure decreased nighttime melatonin plasma levels (Gutiérrez-Pérez et al., 2023). This is important not only because melatonin is a key hormone during pregnancy, regulating various essential processes for fetal development (Reiter et al., 2014), but also because it plays a role in epigenetic regulation (Linowiecka et al., 2023; Ritonja et al., 2022). Maternal glucocorticoids levels resulting from circadian disruption also affect offspring developmental trajectories and health, leading to a higher risk of metabolic and behavioral disorders in later life, possibly through epigenetic mechanisms (Moisiadis and Matthews, 2014; Lehmann et al., 2023). In this regard, maternal circadian disruption due to DLAN exposure, in addition to affecting maternal physiology, may also alter fetal development and germline cells, significantly impacting the epigenetic imprinting of offspring and their health.

The factors responsible for the transmission and establishment of the long-term intergenerational effects of maternal DLAN exposure prior and during pregnancy are not yet known and will be the focus of our future investigations.

## 5 Conclusion

The data presented here have significant implications for understanding how circadian environmental stimuli, such as light at night, can influence the developmental programming of offspring, leading to negative health outcomes later in life. Our study opens the possibility to explore the interplay between epigenetic mechanisms and hormonal pathways that may mediate these effects, contributing to the framework of DOHaD. Increasing exposure to artificial light at night represents an emerging health risk that requires further research and must be considered in the development of prenatal strategies aimed at ensuring a successful pregnancy and preventing the development of diseases in both the mothers and the progeny.

## Data Availability

The raw data supporting the conclusions of this article will be made available by the authors, without undue reservation.
