# Correction: Maternal exposure to dim light at night induces behavioral alterations in the adolescent and adult offspring Wistar rat

**DOI:** 10.3389/fphys.2026.1765993

**Published:** 2026-02-17

**Authors:** Shellye González-González, Mariana Gutiérrez-Pérez, Mara A. Guzmán-Ruiz, Estefania Espitia-Bautista, Rosa María Pavón, Karla P. Estrada-Rodríguez, Alejandro Díaz-Infante R., Cecilia G. Guadarrama Gándara, Carolina Escobar, Natalí N. Guerrero-Vargas

**Affiliations:** 1 Departamento de Anatomía, Facultad de Medicina, Universidad Nacional Autónoma de México, Ciudad de México, Mexico; 2 Department of Psychology, University of Fribourg, Fribourg, Switzerland; 3 Departamento de Fisiología, Facultad de Medicina, Universidad Nacional Autónoma de México, Ciudad de México, Mexico; 4 Instituto Nacional de Psiquiatría, Ramón de la Fuente Muñiz, Ciudad de México, Mexico; 5 Facultad de Arquitectura, Universidad Nacional Autónoma de México, Ciudad de México, Mexico

**Keywords:** circadian disruption, dim light at night, offspring, social play behavior, nucleus accumbens, microglia, depressive and anxiety-like behaviors

There was a mistake in the caption of Figure 3 as published. (B) Representative pattern of a female rat from a mother exposed to dim light at night (DLAN, orange circles; n = 8).

**FIGURE 3:**
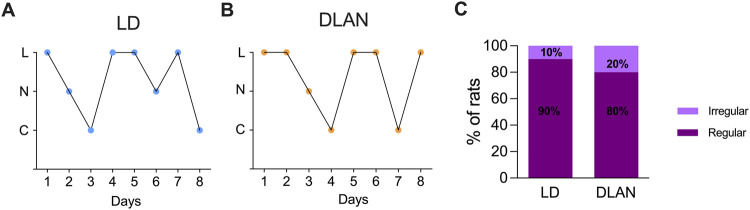
The estrous cycle of the female offspring from mothers exposed to DLAN was not affected. **(A)** Representative graph of the estrous cycle of a female rat from a mother maintained in a regular light-dark cycle (LD, blue circles). **(B)** Representative pattern of a female rat from a mother exposed to dim light at night (DLAN, orange circles). “L” refers to leukocytes, “N” to nucleated cells, and “C” to cornified cells. **(C)** Percentage of rats with regular and irregular estrous cycles in the LD and DLAN groups (n = 10).

The corrected caption of Figure 3 appears below.

(B) Representative pattern of a female rat from a mother exposed to dim light at night (DLAN, orange circles; n = 10).

In the Funding Statement, an incorrect number was provided for DGAPA-PAPIIT. The correct number is DGAPA-PAPIIT IN222524.

A correction has been made to the section [**2 Material and methods**, *2.5 Estrous cycle determination*, Paragraph 1]:

Briefly, a vaginal wash was performed daily at ZT3 over a period of eight days from PN80 to PN88 (n = 10/group).

Regular cycles last from 4 to 5 days (1 proestrus day, 1 estrous day and 1 diestrus I day and 1-2 diestrus II days) and they must have the sequence mentioned before.

The original article has been updated.

