# Supplementary material for: Maternal exposure to dim light at night induces behavioral alterations in the adolescent and adult offspring Wistar rat
Source: Front Physiol. 2025 Jan 7;15:1520160. doi: 10.3389/fphys.2024.1520160 (PMC11747224; doi:10.3389/fphys.2024.1520160)
Supplement: Supplementary file 1 [file Table1.docx]

**Supplementary Figure 1**. Spectral power distribution of lights used for the day light (200-250 lx, 3719K; blue line) and dim light (5-7 lx, 1638K; orange line) during the night phase. Spectral power distribution was measured using a Sekonic C-800 spectrometer (Sekonic,Tokio, Japan).

**Supplementary Figure 2. Maternal DLAN exposure increases body mass in the adolescent male and female offspring. (A)** Body mass of male offspring from mothers maintained in a regular light-dark cycle (LD, blue circles; n=18) and male offspring of mothers exposed to dim light at night (DLAN, orange circles; n= 24) from postnatal day 1 (PN1) to PN25. **(B)** Body mass of male LD and DLAN offspring from PN40 until PN90 (n=13 per group). **(C)** Body mass of female LD (blue circles; n=20) and DLAN offspring (orange circles; n=21) from PN1 to PN25. **(D)** Body mass of female LD and DLAN offspring from PN40 to PN90 (n=9-12/group). Data is shown as the mean + SEM. The Sidak's post hoc test indicated significant differences between groups **p*<0.05, ****p*<0.001.

|  | LD | DLAN | P value |
| --- | --- | --- | --- |
| Total number of pups (n) | 104 | 87 |  |
| Litter size/dam | 10.4 + 1.166 | 8.7 + 1.165 | P=0.31 |
| Body mass of male pups at PN1 | 6.9 + 0.130 | 7.7 + 0.140 | P< 0.0001 |
| Body mass of female pups at PN1 | 6.3 + 0.156 | 7.0 + 0.100 | P= 0.0005 |

**Supplementary Table 1**. Characteristics of male and female offspring of mothers maintained in a regular light-dark cycle (LD) and offspring of mothers exposed to dim light at night (DLAN). The litter seize/dam and body mass of pups at postnatal day 1 (PN1) data are presented as the mean + SEM.

| Behavior | LDm | DLANm | P value |
| --- | --- | --- | --- |
| Carrying | 6.5 (1-25) | 88 (0-20) | P=0.40 |
| Licking | 6 (1-14) | 3 (0-9) | P=0.39 |
| Latency of maternal  behavior | 46.8+ 37.1 | 44.7+ 26.7 | P=0.88 |

**Supplementary Table 2**. **DLAN does not alter maternal behavior.** Maternal behavior directed to the offspring of light-dark cycle mothers (LDm) and dim-light exposure at night mothers (DLANm). For carrying and licking, the data are presented as the median (minimum and maximum). The latency to exhibit maternal behavior is presented as the mean + SEM (n=9-10 per group).
